# Injectable Sodium Hyaluronate Hydrogels Modified by Ionic and Nonionic Polymers Loaded with Prednisolone Disodium Phosphate: Molecular Interactions and Intra-Articular Drug Delivery

**DOI:** 10.3390/ijms27094145

**Published:** 2026-05-06

**Authors:** Dorota Wójcik-Pastuszka, Weronika Pacześniak, Witold Musiał

**Affiliations:** Department of Physical Chemistry and Biophysics, Faculty of Pharmacy, Wroclaw Medical University, ul. Borowska 211A, 55-556 Wroclaw, Poland; dorota.wojcik-pastuszka@umw.edu.pl (D.W.-P.); werapacz@gmail.com (W.P.)

**Keywords:** sodium hyaluronate hydrogels, synthetic polymers, prednisolone disodium phosphate, drug-polymer interactions, intra-articular drug delivery, drug release kinetics

## Abstract

Degenerative joint disease is a major cause of disability, and although glucocorticosteroids and hyaluronic acid are widely used to reduce inflammation and improve joint mobility, the development of effective delivery systems remains a challenge. This study describes injectable sodium hyaluronate (HA)-based hydrogels modified with synthetic polymers, including polyacrylic acid (PA), ammonium acryloyldimethyltaurate/VP copolymer (AX), a polyvinyl acetate–polyvinylpyrrolidone mixture (PVA–PVP), and polyethylene glycol 4000 (PEG), loaded with prednisolone disodium phosphate (PSP). The aim was to investigate molecular interactions between PSP and HA-based polymer networks and to determine how these interactions influence hydrogel structure, viscosity, and drug release. Viscosity was measured using a Brookfield rotational viscometer, while intermolecular interactions were analyzed by ATR–FTIR and DSC. Drug release was evaluated using a paddle-over-disc apparatus and quantified spectrophotometrically. Release kinetics were analyzed using zero-, first-, and second-order models as well as the Higuchi, Korsmeyer–Peppas, and Peppas–Sahlin equations. PSP incorporation affected the dynamic viscosity of all formulations, and excipient type also significantly influenced hydrogel viscosity. ATR–FTIR and DSC analyses indicated hydrogen bond formation between PSP and the macromolecules of HA, PA, AX, and PEG. The PA-containing formulation formed the most extensive polymer network structure and exhibited the highest viscosity. Drug release followed mainly first-order, Higuchi, and Korsmeyer–Peppas models, while the release exponent n (0.58 ± 0.01–0.60 ± 0.01) indicated anomalous transport. These findings provide molecular insight into drug–polymer interactions in HA-based hydrogels and highlight their potential as injectable systems for intra-articular delivery of PSP.

## 1. Introduction

Degenerative joint disease is a common condition characterized by the progressive degeneration of joint cartilage and surrounding tissues. The risk of developing this disorder increases with age, and it is frequently accompanied by pain and restricted joint mobility. These symptoms significantly impair daily functioning, reduce work capacity, and may ultimately lead to disability. The increasing prevalence of joint diseases in aging populations places a considerable burden on healthcare systems, which is further exacerbated by a shortage of specialists, insufficient access to comprehensive geriatric care, and a growing demand for symptomatic treatment. Consequently, there is an urgent need for the development of new, effective, and well-tolerated therapeutic approaches [1,2].

Anti-inflammatory drugs, including corticosteroids and nonsteroidal anti-inflammatory drugs (NSAIDs), are widely used in the treatment of joint inflammation [3,4,5]. Corticosteroids provide rapid relief of pain and inflammation; however, systemic administration is associated with gastrointestinal adverse effects and an increased risk of cardiovascular complications [6,7,8]. These limitations have stimulated growing interest in local drug delivery strategies, particularly intra-articular administration. Intra-articular drug delivery enables the direct administration of therapeutic agents into the joint cavity, allowing high local drug concentrations while minimizing systemic exposure and associated adverse effects [9]. According to Richard et al. [3] and the recommendations of the Osteoarthritis Research Society International (OARSI) [7], intra-articular injections of corticosteroids and hyaluronic acid are among the most commonly used treatments for joint diseases.

**Figure 1:**
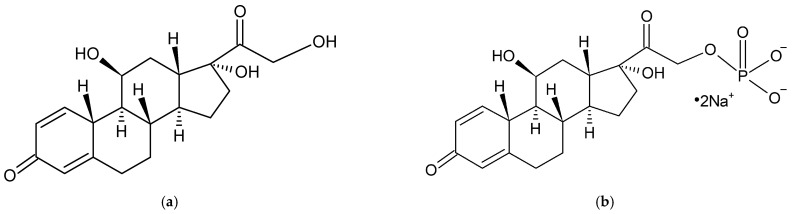
The structural formula of (**a**) prednisolone and (**b**) prednisolone disodium phosphate [13].

Prednisolone is a corticosteroid frequently prescribed for inflammatory conditions affecting the joints and spine, including rheumatoid arthritis, ankylosing spondylitis, bursitis, and synovitis [10]. Its anti-inflammatory activity results from binding to cytoplasmic glucocorticoid receptors and modulating gene expression, leading to inhibition of pro-inflammatory mediators such as prostaglandins and leukotrienes [10,11,12]. The structural formulas of prednisolone and prednisolone disodium phosphate are shown in Figure 1.

In pharmaceutical formulations, prednisolone is available in several salt forms, including disodium phosphate and sodium succinate [10], and is marketed in various dosage forms such as oral liquids, suspensions, tablets, and injectable preparations [14,15,16,17,18].

In recent years, hydrogel-based systems have attracted considerable attention as carriers for intra-articular drug delivery. Hydrogels are three-dimensional polymeric networks capable of absorbing large amounts of water, which provides them with high biocompatibility and the ability to control a drug release [19]. Hyaluronic acid-based hydrogels are particularly promising due to their biocompatibility, biodegradability, and natural occurrence in synovial fluid. Several hydrogel systems have been proposed for the local treatment of joint diseases. For example, Lee et al. [20] developed a hyaluronic acid-based hydrogel conjugated with cyclic inhibitory peptides and a temperature-sensitive polymer for the treatment of rheumatoid arthritis, demonstrating prolonged drug release after intra-articular injection. Similarly, Rouco et al. [21] reported thermosensitive hydrogels designed for the intra-articular delivery of β-lapachone for osteoarthritis therapy.

Hydrogel-based systems can enable molecular-level interactions between the drug and the polymeric matrix, which may influence the physicochemical properties of the formulation as well as drug release behavior. In particular, hydrogen bonding and other non-covalent interactions between the drug molecules and polymer chains can contribute to the formation of a more stable three-dimensional network and modify the rheological properties of the hydrogel. Understanding these interactions is therefore essential for the rational design of hydrogel-based intra-articular drug delivery systems. Despite the growing interest in hydrogel-based intra-articular delivery systems, few studies have systematically investigated how molecular interactions between corticosteroids and the polymeric matrix influence the rheological properties and drug release behavior. A better understanding of these interactions is crucial for optimizing hydrogel formulations for controlled and effective intra-articular therapy [15,22].

The aim of the present study was to develop sodium hyaluronate-based hydrogels modified with ionic or nonionic synthetic polymers, containing prednisolone disodium phosphate intended for intra-articular administration. The interactions between the components of the formulations, as well as their viscometric properties, were investigated. Attenuated Total Reflectance—Fourier Transform Infrared Spectroscopy (ATR–FTIR), Differential Scanning Calorimetry (DSC) and rotational viscosity measurements were applied. Furthermore, the release behavior of prednisolone disodium phosphate from the prepared hydrogels was evaluated to determine the potential of the developed systems for intra-articular drug delivery.

## 2. Results and Discussion

### 2.1. Viscosity Investigation

**Figure 2:**
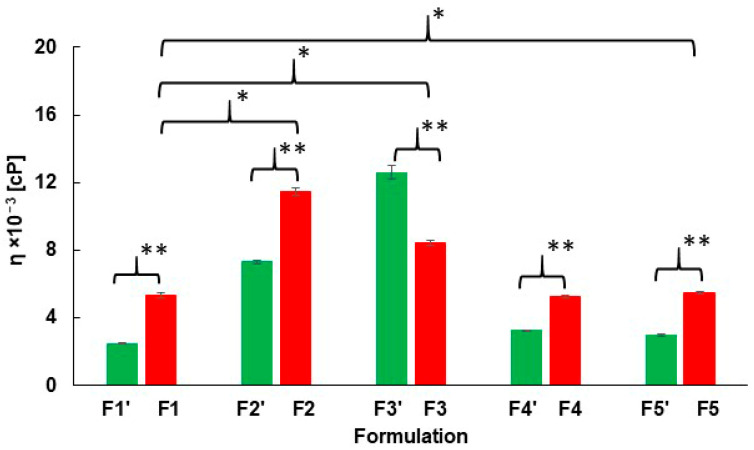
Dynamic viscosities of hydrogels F1–F5 (red) and their drug-free counterparts F1’–F5’ (green) [23], *n* = 6, *p* < 0.05. A single asterisk above a bar indicates a significant difference due to the addition of an auxiliary polymer, whereas a double asterisk indicates a difference resulting from the addition of PSP.

The results of the viscosity investigation are shown in Figure 2. The dynamic viscosities of hydrogels F1–F5 (composition presented in Table 1), indicated by the red columns, differed, as confirmed by Student’s *t*-test.

Statistically significant differences were observed between formulation F1 and hydrogels F2, F3, and F5, indicated in the figure by bars with a single asterisk. In contrast, no significant difference was found between F1 and F4, suggesting that the PVA–PVP mixture did not influence the dynamic viscosity of the preparation. The largest differences were observed between F1 and F2, doped with PA, and between F1 and F3, incorporated with AX. Dynamic viscosity values of these compositions without PSP (F1’–F5’) were presented in our previous study [23] showing a similar trend: F1’ had the lowest viscosity, whereas hydrogels containing the auxiliary polymers exhibited higher values. The viscosities of F1’–F5’ are also presented in Figure 2 and indicated in green columns. These results show that the addition of polymers, particularly PA and AX, increases the dynamic viscosity of the hydrogels, as discussed in our previous study [23].

The influence of PSP incorporation into carriers F1–F5 on hydrogel viscosity was investigated. The addition of the drug increased the viscosity of hydrogels F1, F2, F4 and F5, whereas a decrease in dynamic viscosity was observed for hydrogel F3. These observations suggest possible interactions between PSP and the polymers used. However, no direct evidence or detailed mechanistic description of a chemical or pharmacological interaction between HA and PSP has been reported in the literature.

Under physiological conditions, HA is a hydrophilic, anionic polymer due to the presence of COO^−^ and OH^−^ groups. PSP is a water-soluble, hydrophilic molecule containing negatively charged phosphate groups (–PO_4_^3−^), but also possesses a hydrophobic core. Direct ionic interactions between HA and PSP are unlikely because the like charges repel each other. However, indirect interactions mediated by Na^+^ ions may occur. Na^+^ can form ionic bridges between HA and PSP, increasing the viscosity of the compositions. In addition, in the presence of PBS or other salts, anion–anion repulsion is reduced, allowing HA chains to approach PSP more closely [24].

Hydrogen bonding is another potential mechanism contributing to viscosity enhancement. Both HA and PSP contain polar groups capable of forming hydrogen bonds with each other and with water molecules. When mixed, a dense network of hydrogen bonds may form, restricting molecular mobility and increasing the effective viscosity. Moreover, solvation of PSP through hydrogen bonding, dipole–dipole, or ion–dipole interactions can create a stable solvation shell around the drug, increasing its hydrodynamic volume and further enhancing the viscosity of the hydrogel [25]. Analogies from the literature support the potential for such interactions. Balima et al. [26] reported that HA/chitosan hydrogels with higher HA content exhibited enhanced mechanical properties, partly due to hydrogen bonding and, in the presence of Zn^2+^ ions, crosslink formation between network chains. Ewurum et al. [27] demonstrated that hydrophobic patches on HA can interact with lipids in aqueous solution. By analogy, the hydrophobic core of PSP may interact with these regions on HA, providing additional “anchor points” that stiffen the network, reduce interchain distances, and contribute to physical crosslinking.

The observed changes in viscosity following PSP incorporation are likely the result of multiple, synergistic interactions: ionic bridges mediated by Na^+^, hydrogen bonding networks, dipole interactions, solvation effects, and potential hydrophobic interactions between the HA and PSP molecules. These combined mechanisms lead to a denser, more ordered, and mechanically reinforced hydrogel network.

Formulation F2 was doped with PA. This macromolecule, similarly to HA, contains carboxyl groups in its structure that are responsible for its hydrophilic properties. These functional groups are capable of forming hydrogen bonds with water, engaging in dipole–dipole interactions, and, in their ionic form (–COO^−^), participating in ion–dipole interactions with water molecules [28]. Therefore, it can be expected that PSP interacts with PA in a manner analogous to its interaction with HA, and these interactions may explain the observed increase in the viscosity of formulation F2 following drug incorporation. Furthermore, Bayliss et al. [29] investigated hydrophilic polymers such as PA, PVA, PVP, and PEG, demonstrating that PVA, PVP, and PEG exhibit behavior similar to that of PA. This suggests that the increase in dynamic viscosity observed in formulations F4 and F5 after PSP incorporation may be governed by mechanisms similar to those operating in formulations F1 and F2. Surprisingly, formulation F3 exhibited a decrease in dynamic viscosity following PSP incorporation. This behavior may be attributed to the presence of AX, which appears to alter the interaction pattern between the polymeric matrix and the incorporated drug. AX is a hydrophilic anionic copolymer containing sulfonate groups that ensure strong hydration and water solubility. However, due to its distinct chemical structure and the presence of permanently ionized –SO_3_^−^ groups, its interaction pattern with drug molecules differs from that observed for carboxylate-based polymers such as PA or HA. Gouveia et al. [30] studied the viscosity of formulations containing carbamide peroxide with PA and AX and found that the viscosity of the gel containing PA was higher than that of the formulation containing AX, which is consistent with the findings of the present study. Gao et al. [31] demonstrated that viscosity depends on the hydrogen-bond network. Certain ions and molecules can disrupt the ordered hydrogen-bond network of water, making it less structured. This increased disorder enhances molecular mobility, weakens hydrogen bonding between water molecules, and ultimately reduces viscosity. This phenomenon may explain the decrease in dynamic viscosity observed in formulation F3 after PSP incorporation. Therefore, the observed viscometric differences between the formulations appear to be primarily governed by the specific intermolecular interactions between PSP, the polymer matrix, and the surrounding water molecules.

Furthermore, the pH values of formulations F1–F5 were measured and the obtained results are as follows: 7.62 ± 0.04, 4.73 ± 0.05, 7.61 ± 0.02, 7.29 ± 0.08, and 7.56 ± 0.02, respectively. The observed differences, particularly the lower pH of F2, may influence the degree of ionization and also contribute to variations in viscosity [32].

### 2.2. ATR–FTIR Study

**Figure 3:**
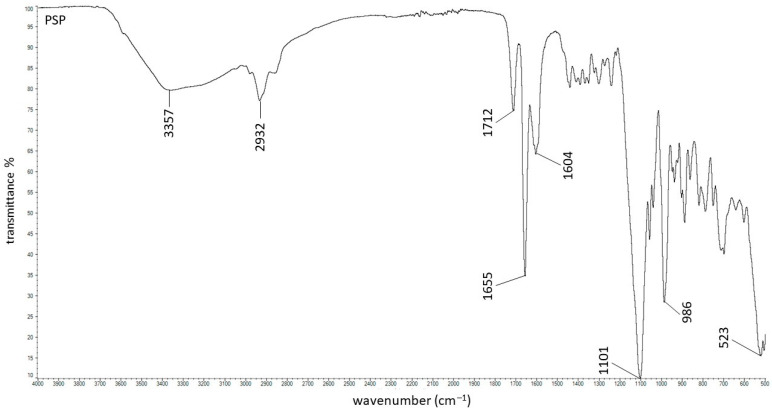
The ATR–FTIR spectrum of PSP.

The structures and ATR–FTIR spectra of all polymers, as well as interactions between the macromolecules used in the study have already been presented in previous works [23,33,34]. In the present study, we focused on the interactions between the drug and the carrier. The spectrum of pure PSP is presented in Figure 3.

The broad band at 3357 cm^−1^ was assigned to hydrogen-bonded stretching vibrations of the –OH group. The band at 2932 cm^−1^ corresponds to C–H stretching vibrations. The characteristic signals of PSP were observed at 1712 cm^−1^ and 1655 cm^−1^, attributed to C=O stretching vibrations. Additional signals at 1604 cm^−1^, and 986 cm^−1^ were also observed and the bands correlate well with literature data [35]. Smith [36] reported a strong peak of P–O stretching from –PO_4_^3−^ group at 1047 cm^−1^ which typically may be observed between 1000 and 1100 cm^−1^ on the FTIR spectra. Additionally, the signal of P–O bending vibrations from phosphate group is usually visible between 500 and 600 cm^−1^ [37]. In the spectrum shown in Figure 3, the bands corresponding to P–O stretching and bending vibrations of the –PO_4_^3−^ group were observed at 1101 cm^−1^ and 523 cm^−1^, respectively.

**Figure 4:**
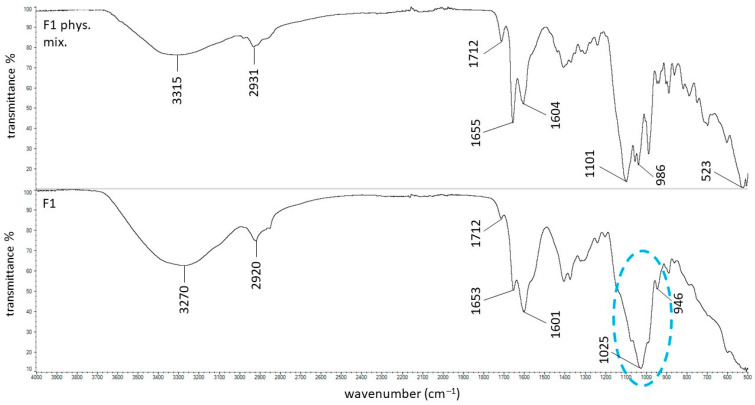
The ATR–FTIR spectra of the F1 physical mixture and the F1 formulation; the changes between these spectra were marked in blue.

The ATR–FTIR spectra of the physical mixture of PSP and HA, as well as the F1 formulation composed of PSP, HA, and water, are presented in Figure 4.

It can be noted that all characteristic bands of PSP were observed in the spectrum of the F1 physical mixture. However, in the spectrum of the F1 formulation, the band at 1101 cm^−1^, attributed to the P–O stretching vibrations of PSP, was shifted to 1025 cm^−1^, suggesting intermolecular interactions between PSP, HA, and water. Such a shift toward lower wavenumbers may result from hydrogen-bond formation involving the phosphate groups, which weakens the P–O bond and decreases its vibrational frequency. The phosphate group (–PO_4_^3−^ or its derivatives) contains highly electronegative oxygen atoms with lone pairs of electrons, which can act as hydrogen bond acceptors and form hydrogen bonds with water molecules. Additionally, HA contains –OH and –NHCO– groups, which can be hydrogen bond donors and form hydrogen bonds with the phosphate groups of PSP [38,39]. The interactions between the phosphate groups of PSP, water molecules, and HA explain the band shift from 1101 cm^−1^ to 1025 cm^−1^. These changes are indicated in blue in Figure 4. PSP also contains hydroxyl groups (Figure 1), and their stretching vibrations were observed at 3357 cm^−1^ in the spectrum of PSP. In the spectrum of the F1 physical mixture, this band was observed at 3315 cm^−1^, while in the spectrum of the F1 formulation it became broadened and more intense. These changes in the position and intensity of the O–H bands indicate the formation of hydrogen bonds between PSP and HA. Hydroxyl groups from PSP may interact with the –COO^−^ groups of HA, forming O–H⋯O^−^ hydrogen bonds (hyaluronan carboxylate), as well as with –OH and –NHCO– groups of HA, forming O–H⋯O hydrogen bonds (hydroxyl or amide) [40,41,42,43].

In a hydrated environment, hydrogen bonds may form between PSP, HA, and surrounding water molecules, which contributes to the stabilization of the system. These findings are consistent with the viscosity data, indicating that the incorporation of PSP into the HA hydrogel increases its viscosity due to the formation of an internal hydrogen-bonding network.

**Figure 5:**
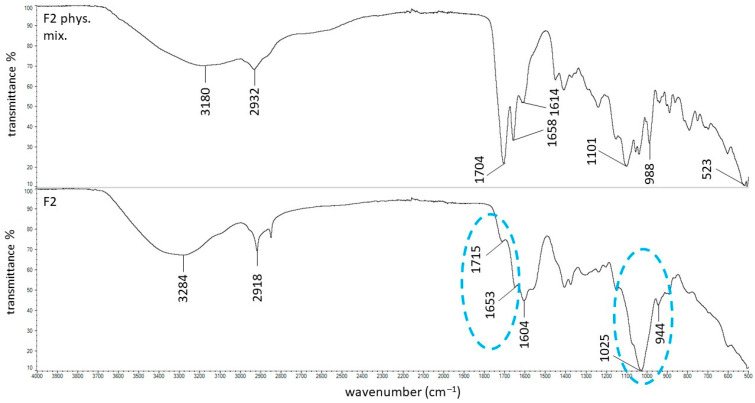
The ATR–FTIR spectra of the F2 physical mixture and the F2 formulation; the changes between these spectra were marked in blue.

A shift and deepening of the PSP phosphate group band at 1101 cm^−1^ was also observed in formulation F2, confirming interactions between the drug and HA. Formulation F2 was doped with the auxiliary polymer PA, whose very strong and sharp band corresponding to the carboxyl group is visible in the spectrum of the physical mixture F2 (Figure 5) at 1704 cm^−1^. In this region, the characteristic bands of PSP and HA are not distinguishable. In contrast, the spectrum of formulation F2 shows only a very weak band at 1715 cm^−1^, which can be attributed to the carboxyl groups of HA and PSP, while the intense band at 1704 cm^−1^ disappears. This shift toward a higher wavenumber likely results from changes in the local hydrogen-bonding environment of the carboxyl groups after incorporation into the hydrated polymer network, indicating the formation of hydrogen bonds between the carboxyl group of PA (acting as a hydrogen-bond acceptor) and the hydroxyl groups of PSP (acting as hydrogen-bond donors) [44]. The formation of these additional hydrogen bonds between PA and PSP likely contributes to the increased dynamic viscosity of formulation F2, which is significantly higher than that of F1, as they reinforce the internal intermolecular network within the hydrogel. These spectroscopic observations are consistent with the viscometric data, confirming that strengthened hydrogen-bonding interactions play a key role in viscosity enhancement.

**Figure 6:**
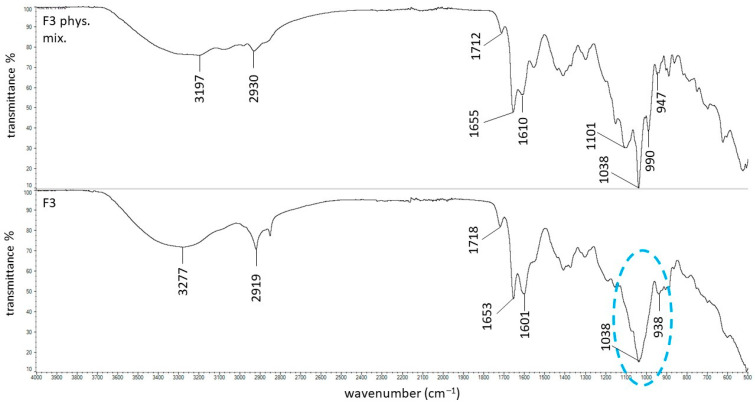
The ATR–FTIR spectra of the F3 physical mixture and the F3 formulation; the changes between these spectra were marked in blue.

The ATR–FTIR spectra of the physical mixture of F3 and formulation F3 are presented in Figure 6. In this case, the band observed in the spectrum of the physical mixture at 1101 cm^−1^, corresponding to the phosphate group of PSP, is absent in the spectrum of formulation F3, suggesting that the local environment of the phosphate group is altered upon incorporation into the polymer matrix. Furthermore, the sharp band at 1038 cm^−1^ observed in the physical mixture, attributed to AX, becomes broadened and less intense in the spectrum of formulation F3. This broadening indicates the formation of a dynamic hydrogen-bonding network between PSP and AX, reflecting multiple interactions with the sulfonate and carbonyl groups of the polymer. PSP contains hydroxyl groups (–OH) that can act as hydrogen-bond donors, while AX possesses hydrogen-bond acceptors such as sulfonate (–SO_3_^−^) and carbonyl (C=O) groups from vinylpyrrolidone units. In this context, the hydroxyl groups of PSP form noncovalent hydrogen bonds with the electronegative oxygen atoms in the polymer’s acceptor sites (O–H⋯O=S, O–H⋯O=C) [45]. These observations confirm the presence of molecular interactions between PSP and AX; however, they do not necessarily imply strengthening of the polymer network. Rheological data indicate that the presence of PSP leads to a decrease in viscosity (F3 vs. F3’), suggesting that PSP–polymer interactions may partially compete with or disrupt polymer–polymer interactions within the AX network, resulting in reduced chain entanglement or network cohesion. Accordingly, the increase in viscosity observed between F1 and F3 should be primarily attributed to the intrinsic rheological properties of AX. Nevertheless, the viscosity of F3 remained lower than that of formulation F2 doped with PA, which exhibited the highest viscosity among all formulations. This suggests that the interactions involving PA are either stronger or more numerous, resulting in a more extensive hydrogen-bonding network and greater resistance to flow. These spectral changes indicate that the interactions between PSP and AX, mediated by hydrogen bonding, contribute to the internal organization of the hydrogel, although their net effect is modulated by competing interactions within the system, ultimately influencing its viscometric behavior.

**Figure 7:**
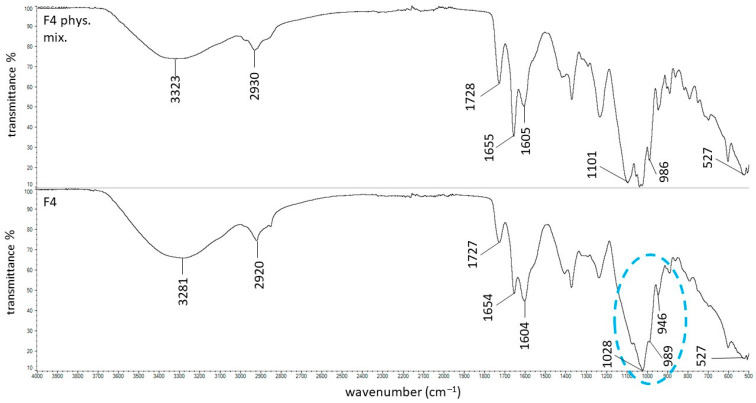
The ATR-FTIR spectra of the F4 physical mixture and the F4 formulation; the changes between these spectra were marked in blue.

The ATR–FTIR spectra of the physical mixture F4 and formulation F4 are shown in Figure 7. Spectral differences between the physical mixture and the final formulation were observed in the 1000–1100 cm^−1^ region and in the 3000–3500 cm^−1^ range. However, these changes were identical to those previously observed between the spectrum of the F1 physical mixture and the F1 formulation, which were attributed to the formation of hydrogen bonds between PSP and HA. Consequently, the ATR–FTIR spectra provide no evidence for the formation of additional hydrogen bonds between PSP and the PVA–PVP mixture. This observation is fully consistent with the viscometric results: the dynamic viscosities of formulations F1 and F4 were identical, and statistical analysis revealed no significant differences between their values. The absence of viscosity enhancement in formulation F4 further supports the spectroscopic findings and confirms that the PVA–PVP mixture does not contribute to the strengthening or expansion of the hydrogen-bonding network within the hydrogel. These results indicate that while PSP engages in hydrogen bonding with HA, the PVA–PVP mixture in F4 does not form additional interactions, which explains the lack of viscosity increase in this formulation after PVA–PVP addition.

**Figure 8:**
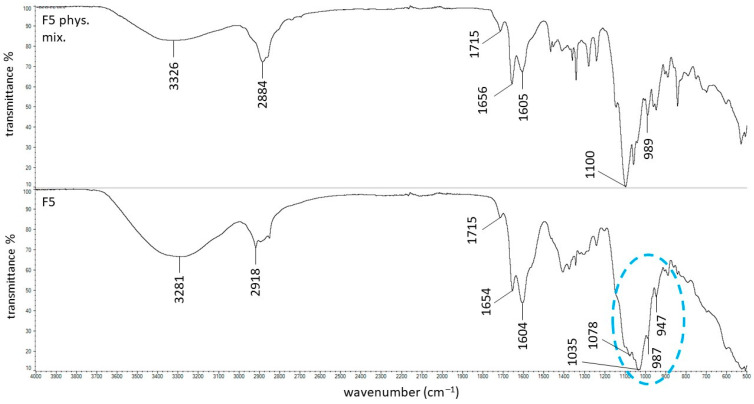
The ATR-FTIR spectra of the F5 physical mixture and the F5 formulation; the changes between these spectra were marked in blue.

Figure 8 presents the ATR–FTIR spectra of the F5 physical mixture and formulation F5. This formulation was doped with PEG, which exhibits a strong, sharp band at 1100 cm^−1^, similar to PSP. This signal is clearly visible in the spectrum of the F5 physical mixture; however, in the spectrum of the F5 formulation, a very weak signal at 1078 cm^−1^ and a broadened maximum at 1035 cm^−1^ are observed. Both the oxygen atoms of the phosphate group (P=O and –O^−^) and the oxygen atoms in the ether groups (–CH_2_–CH_2_–O) of PEG act as hydrogen-bond acceptors, indicating that direct donor–acceptor hydrogen bonding between PEG and the phosphate group is unlikely. However, Petra et al. [46] demonstrated that water molecules in the vicinity of PEG chains are highly ordered and form extensive hydrogen-bond networks in the first and subsequent hydration shells, highlighting the role of water as an intermediary in interactions involving PEG. Additionally, Yang et al. [47] reported that each PEG unit can bind water directly through hydrogen bonds and that, in the presence of ions (e.g., in phosphate buffers), multilayer water shells are formed, mediating interactions between PEG and ions or other polar groups. Based on these studies, it can be postulated that the phosphate group of PSP may form indirect hydrogen bonds with PEG via water molecules, as illustrated by the interaction: PEG–O⋯H–O–H⋯O–PO_4_^3−^. Molecular dynamics and structural studies have shown that water-mediated hydrogen bonds–where a single water molecule bridges two polar groups—generally exhibit lower occupancy and shorter lifetimes than direct donor–acceptor hydrogen bonds, indicating that water-bridged interactions are weaker and more dynamic than conventional hydrogen bonds [48,49,50]. These weak, water-mediated interactions may account for the slight increase in the dynamic viscosity of formulation F5 compared with F1. In formulation F5, in addition to the hydrogen-bonding interactions already present in F1, additional hydrogen bonds are formed via water bridges between PEG and PSP. Although individually weak, the cumulative and cooperative nature of these indirect interactions may modestly restrict molecular mobility, resulting in a small but measurable increase in viscosity.

To sum up, the ATR–FTIR analysis reveals that PSP interacts with hydrogel polymers primarily through hydrogen bonding. In formulations containing HA or PA, direct hydrogen bonds between PSP hydroxyl groups and polymer carboxyl, hydroxyl, or amide groups strengthen the internal network, leading to increased viscosity. PSP also forms direct hydrogen bonds with AX, slightly enhancing viscosity, while no additional hydrogen bonding is observed with the PVA–PVP mixture, consistent with unchanged viscosity. In PEG-containing formulations, PSP engages in indirect, water-mediated hydrogen bonds, which are weaker than direct interactions but still produce a slight viscosity increase. These results highlight that both direct and water-bridged hydrogen bonds play key roles in stabilizing the drug–polymer system and modulating its rheological properties.

It should be noted that ATR–FTIR spectra were acquired from dried samples, which may not fully reflect the interaction mechanisms present in hydrated hydrogel systems. This is particularly relevant for formulation F5, containing prednisolone sodium phosphate (PSP), sodium hyaluronate, and PEG. Water plays a critical role in this system by influencing hydrogen bonding, ionic interactions, and polymer chain conformation. In the hydrated state, water molecules may compete with PSP for interaction sites on sodium hyaluronate or PEG, or alternatively act as a mediator facilitating intermolecular associations. Additionally, the high hydration level of sodium hyaluronate significantly affects its structural organization and interaction potential. Drying may lead to structural rearrangements and enhanced intermolecular interactions that are less pronounced under hydrated conditions. Therefore, the ATR–FTIR results should be interpreted as indicative of potential interaction sites and tendencies rather than a direct representation of the system under conditions used for rheological and drug release studies. Despite this limitation, the observed correlations between spectroscopic results and macroscopic behavior suggest that the identified interactions may remain relevant, although their nature and strength could differ under hydrated conditions.

### 2.3. DSC Analysis

**Figure 9:**
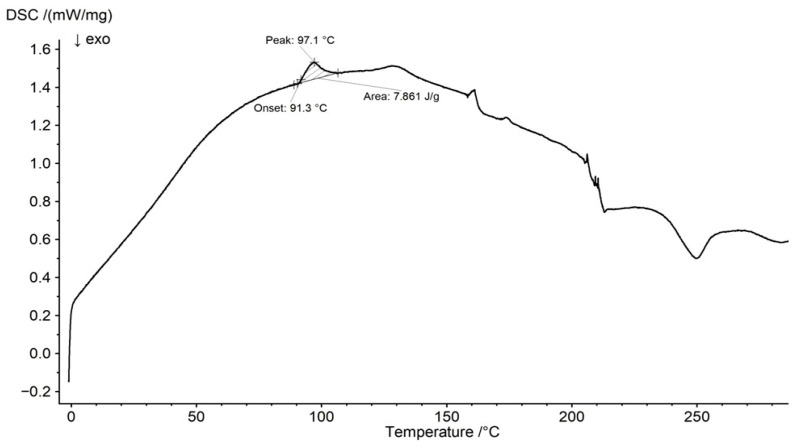
DSC thermogram of PSP.

**Figure 10:**
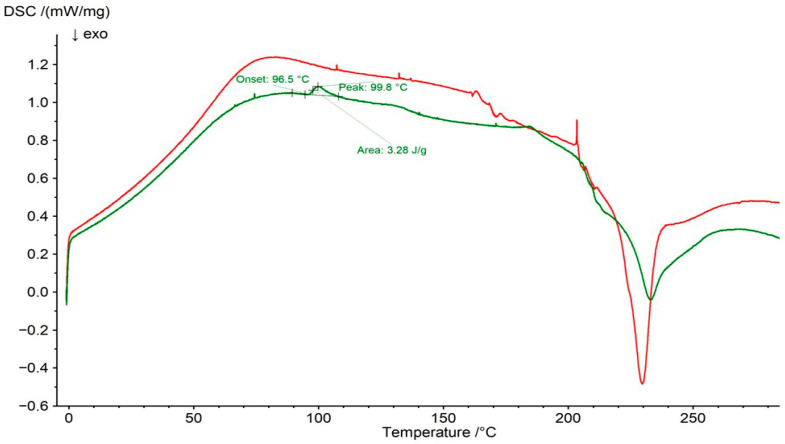
DSC thermogram of F1 physical mixture (green line) and formulation F1 (red line).

The DSC thermogram of pure PSP is presented in Figure 9. The melting peak was observed at 97.1 °C, whereas Manandhar et al. [51] reported it at 118.18 °C. This endothermic peak was also visible in the DSC curve of the F1 physical mixture at 99.8 °C, with an onset temperature of 96.5 °C (Figure 10). Its area was lower than that observed for pure PSP, which may result from the addition of the polymer to the mixture and indicates the absence of significant interactions between PSP and water. However, in the DSC thermogram of the F1 formulation (Figure 10), this peak was no longer detectable, suggesting interactions between PSP and HA. This observation is consistent with the ATR–FTIR results, which indicated the formation of a common hydrogen-bonding network among PSP, HA, and water. Similarly, Lin et al. [52] demonstrated that hydrogen-bonding interactions between drugs and polymers can affect drug miscibility and stability, with the disappearance of melting peaks and shifts in the glass transition temperature (T_g_) serving as evidence of such interactions.

**Figure 11:**
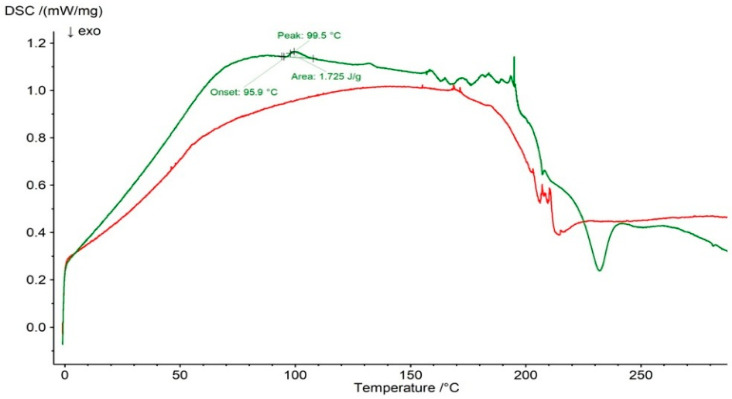
DSC thermogram of F2 physical mixture (green line) and formulation F2 (red line).

Comparable results were obtained for the F2 formulation. The characteristic endothermic peak was present in the DSC curve of the F2 physical mixture but disappeared in the thermogram of the F2 formulation (Figure 11), suggesting the formation of a hydrogen-bonding network between PSP and both polymers, as confirmed by ATR–FTIR analysis. Medarević et al. [53] noted that the absence of characteristic melting peaks in DSC thermograms may indicate that the drug is present in an amorphous state or is molecularly dispersed within the polymer matrix. They also reported that shifts in T_g_ and changes in heat capacity (C_p_) can be interpreted as evidence of molecular miscibility and drug–polymer interactions.

**Figure 12:**
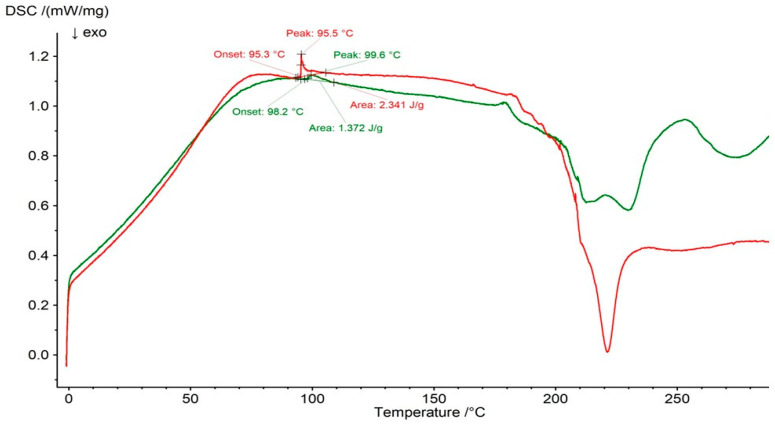
DSC thermogram of F3 physical mixture (green line) and formulation F3 (red line).

It should be noted that the main melting peak of PSP was present in both the DSC thermogram of the F3 physical mixture and the DSC curve of the F3 formulation (Figure 12). However, slight differences were observed in the peak temperatures, onset temperatures, and peak areas, which may result from interactions between the drug and the polymer.

**Figure 13:**
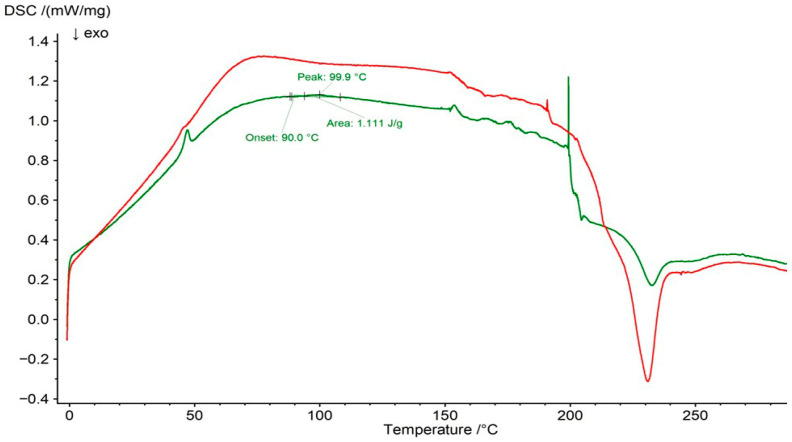
DSC thermogram of F4 physical mixture (green line) and formulation F4 (red line).

The melting peak of PSP was observed at 99.9 °C in the DSC thermogram of the F4 physical mixture (Figure 13) but was absent in the DSC curve of the F4 formulation (Figure 13). Budiman et al. [54] investigated the interaction between alpha–mangostin (AM) and PVP and reported that the endothermic melting peak of AM was not detected in the DSC curve of the AM–PVP system. They postulated that AM became amorphous in the presence of PVP and that interactions between AM and PVP occurred, effectively entrapping the drug within the polymer matrix. FTIR analysis further revealed intermolecular interactions between AM, acting as a proton acceptor, and PVP, acting as a proton donor. In the present study, the absence of the PSP melting peak in the DSC thermogram of the F4 formulation may be attributed to its amorphization rather than hydrogen-bond formation, as ATR–FTIR analysis did not provide evidence of hydrogen-bonding interactions between the drug and the polymers [54].

**Figure 14:**
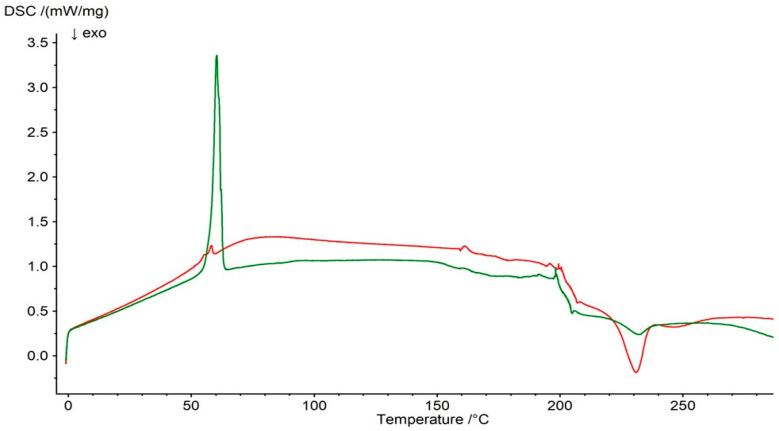
DSC thermogram of F5 physical mixture (green line) and formulation F5 (red line).

In the DSC thermogram of the F5 physical mixture (Figure 14), the strong, sharp PEG signal at 60.4 °C partially overlapped and flattened the PSP peak at 97.1 °C. The PSP endothermic peak was also absent in the DSC curve of the F5 formulation (Figure 14). However, this absence is unlikely to result from the presence of the PEG peak, as the PEG signal in the formulation was considerably smaller. The notable decrease in the intensity of the PEG peak in the F5 formulation may indicate interactions between PEG and PSP, potentially through hydrogen bonding. Therefore, the disappearance of the PSP peak is more likely due to the amorphization of PSP, similar to that observed in the F4 formulation and as reported by Budiman et al. [54], or to interactions between PSP and PEG, as also suggested by ATR–FTIR analysis.

In brief, the DSC results indicate that the melting behavior of PSP is strongly influenced by its interactions with the polymers and its physical state within the formulations. Direct interactions with HA and PA, as observed in F1 and F2, lead to the disappearance of the PSP melting peak, consistent with hydrogen-bond formation, whereas in F3, the PSP peak remains, suggesting weaker or minimal interactions with AX. In F4 and F5, the absence of the PSP peak is likely due to amorphization or water-mediated interactions with PEG, in line with ATR–FTIR findings. These observations demonstrate that both polymer type and the nature of drug–polymer interactions critically affect the thermal properties and physical state of PSP in hydrogel formulations.

### 2.4. Release Study

The present study used phosphate-buffered saline (PBS) as a release medium, which represents a simplified system compared to synovial fluid. Synovial fluid is a complex biological environment containing proteins, hyaluronic acid, and other macromolecules that may influence drug diffusion and release behaviour. Therefore, the obtained results should be interpreted as an initial in vitro approximation of the release profile under controlled conditions.

**Figure 15:**
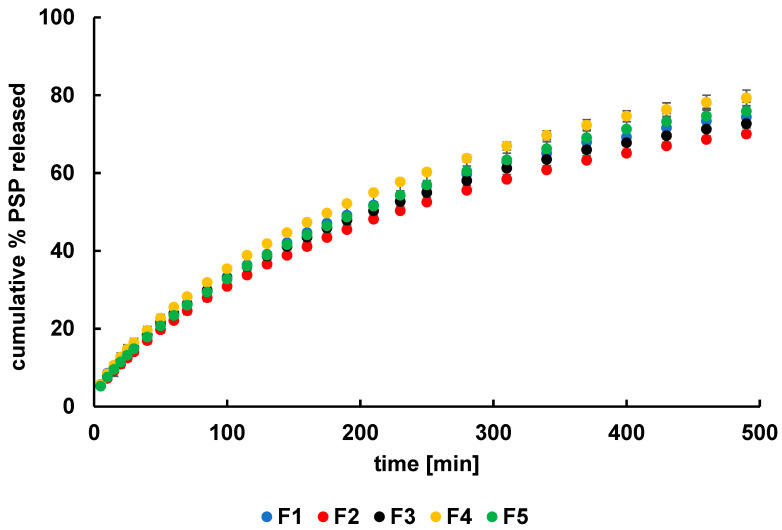
The release profiles of PSP from formulations F1–F5, *n* = 6.

The release profiles of PSP from the obtained formulations are shown in Figure 15. The amounts of drug released within 8 h were 74 ± 2%, 70 ± 1%, 73 ± 2%, 79 ± 2%, and 76 ± 2% from F1, F2, F3, F4, and F5, respectively. Overall, the release curves were very similar, despite differences in hydrogel composition. Although ATR–FTIR and DSC studies indicated interactions between PSP and the polymers, it might be expected that such interactions—particularly with synthetic polymers—would slow the drug release.

The comparison between the dissolution profiles was performed by calculating the difference factor and the similarity factor and the obtained results are listed in Table 2. The values of f_1_ were below 15 and simultaneously the values of f_2_ were above 50 indicating similarity of the release curves [55]. This results mean that the incorporation of the auxiliary polymer did not influenced the PSP release. ANOVA analysis, as well as Student’s *t*-test, confirmed the lack of differences between F1 profile and the curves of PSP release from F2–F5. The possible explanation of the obtained dissolution results may be that the drug release from the investigated formulations is governed predominantly by diffusion through the highly hydrated network of HA rather than by chemical differences between the individual hydrogels.

Drug release from hydrogel systems is not governed by viscosity alone. According to the literature, the dominant factors controlling release include the effective mesh size of the polymer network, diffusion coefficients, and drug–polymer interactions, rather than bulk rheological properties. In particular, for small and highly water-soluble molecules such as PSP, diffusion through hydrated polymer matrices is typically rapid and primarily governed by Fickian transport [56]. Even in more viscous systems, if the polymer network structure does not undergo substantial changes at the microscopic scale, the diffusion pathway for the drug remains largely unaffected. This phenomenon has been widely reported in hydrogel-based delivery systems, where viscosity does not necessarily correlate with release kinetics when the drug is low molecular weight and hydrophilic. Therefore, the similar release profiles (f_2_ > 50) observed for F1 and F2 suggest that viscosity differences were not sufficient to significantly alter the effective diffusion barrier in the system [57].

It should also be noted that the relatively high ionic strength of the release medium may contribute to charge screening within the hydrogel network, reducing the sensitivity of the system to compositional differences and leading to comparable release profiles across all formulations [58].

Although no statistically significant differences were observed, formulation F2 showed a slight tendency toward slower release compared to the other systems, as reflected by marginally lower release profiles and the highest f_1_/lowest f_2_ values in comparison with F1. However, this tendency was not consistent across different kinetic models and remained within experimental variability, and therefore should be interpreted as a trend rather than a meaningful effect.

In particular, the t_0.5_ values derived from different kinetic models did not show a consistent or clinically relevant difference between F1 and F2, further supporting the lack of a meaningful distinction between the formulations.

Overall, these findings indicate that the observed intermolecular interactions, while present at the molecular level, are not sufficiently strong to induce substantial changes in macroscopic drug diffusion under the studied conditions.

Previous studies have reported similar behavior in hydrogel-based delivery systems using the same polymeric carrier, where incorporation of different active pharmaceutical ingredients, including diclofenac sodium, influenced release kinetics only to a limited extent [59]. Paradiso et al. [60], investigating chlorhexidine (CLX) release from hydrogels, reported that CLX—a hydrophilic drug—diffuses more readily through hydrogel networks with a more open structure, characterized by lower crosslinking density and higher water content. Their findings demonstrated that increasing the crosslinker content significantly reduced the release rate of CLX. These results highlight the importance of hydrogel mesh size in controlling drug release, as a more highly crosslinked network reduces water content and introduces steric hindrance to drug diffusion. Thang et al. [61] further reported that interpenetrating polymer networks (IPNs), due to their unique structure and properties, enable controlled and sustained drug release. Additionally, incorporation of different polymers into polymer networks may improve biocompatibility, mechanical strength, stability, and permeability. Considering these findings further studies exploring higher PA concentrations may be considered to investigate whether more pronounced changes in network density could influence release kinetics. Such optimization could be clinically relevant, particularly for patients who cannot use NaDic-loaded hydrogels and may benefit from an alternative PSP-based formulation.

### 2.5. Kinetic Analysis

**Figure 16:**
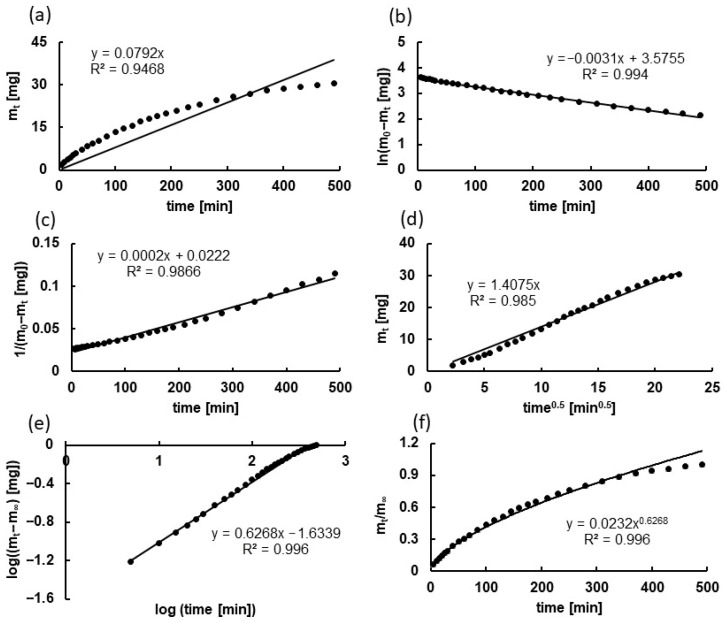
Theoretical curves (solid line ―) fitted to experimental points (dots •) based on PSP release from F1 based on (**a**) zero-order kinetics, (**b**) first-order kinetics, (**c**) second-order kinetics, (**d**) Higuchi model, (**e**) Korsmeyer-Peppas model, (**f**) Peppas-Sahlin model.

Determining the kinetic equations of drug release enables a better understanding and prediction of how the drug is released from the carrier. This information allows for the optimization of the carrier, for example, to achieve a gradual drug release over several days rather than an initial burst (“burst release”). Kinetic analysis also facilitates the prediction of drug concentrations over time at the target site, which is crucial for selecting the appropriate dose and dosing frequency [57,62,63,64]. However, kinetic modeling should be regarded primarily as a descriptive tool rather than definitive evidence of a single transport mechanism. An example of the kinetic analysis of the PSP release from one of the F1 formulation series is shown in Figure 16, and the corresponding kinetic parameters are summarized in Table 3.

The values of the kinetic parameters of the drug dissolution, such as the release rate constants and half release times, were comparable across all formulations for all models. This is consistent with the results of comparison of the release profiles, which are similar. The calculated coefficients of determination R^2^ were high in all employed equations, including zero-order, first-order, Higuchi, and Korsmeyer–Peppas equations, indicating that multiple models can adequately describe the release behavior. The Korsmeyer–Peppas exponent values (*n*) ranged from 0.58 ± 0.01 to 0.60 ± 0.01, suggesting a release behavior consistent with a combination of diffusion-driven transport and additional matrix-related contributions. However, due to the similarly high goodness-of-fit values obtained for different models, no single kinetic model can be unambiguously assigned as the exclusive description of the release mechanism. Complementary analysis using the Peppas–Sahlin equation further supported the predominance of diffusion-related transport, while also indicating that matrix relaxation effects may contribute to the overall release behavior. Nevertheless, these contributions should be interpreted cautiously, as kinetic models in this study serve primarily to describe release trends rather than to definitively resolve mechanistic pathways.

The good fit to the Higuchi model suggests that diffusion through the hydrated matrix plays a substantial role in PSP transport. Therefore, while diffusion appears to be the primary driving force, matrix relaxation may also contribute to the overall release behavior and modulate the kinetics. Although the applied excipients modify the viscometric properties of the formulations, they do not appear to substantially alter the transport-relevant network characteristics, resulting in comparable release behavior among all tested systems. Overall, PSP release appears to be largely governed by diffusion through the highly hydrated hydrogel network, which is consistent with the physicochemical properties of PSP as a small, highly water-soluble, ionized molecule with limited affinity for the polymer matrix [65,66,67,68].

The sustained release effect can be achieved by reducing the effective diffusion coefficient (D_eff_), according to Fick’s law [66]:

$$J=-D_{\mathrm{eff}}\frac{\mathrm{dC}}{\mathrm{dx}}$$

Slowing down the drug release requires a decrease in D_eff_, which can be accomplished by increasing the polymer network density, reducing pore size, limiting the degree of hydration, and increasing the tortuosity of the diffusion pathway. In practice, a significant prolongation of release is observed only when the polymer concentration is markedly increased, the network is partially crosslinked, or swelling is restricted. For sodium hyaluronate-based hydrogels, this would involve substantially increasing the HA concentration (e.g., 2–3-fold), introducing chemical crosslinking, and reducing the extent of network hydration. Similar strategies have been demonstrated by Tsai et al. [69] who investigated prednisone-loaded HA hydrogels crosslinked with dithiothreitol (DTT) and incorporating nonphospholipid liposomes. Their integrated system enabled sustained release of prednisolone over an extended period, with release kinetics tunable by adjusting the crosslinking density of the HA network, demonstrating a versatile platform for controlled and prolonged drug release. In the present study, the addition of the excipient to the hydrogel did not result in a significant modification of the drug release. Nevertheless, it is well established that modified polymer networks can substantially alter mechanical properties, increased viscosity, and stability, which in turn may modulate the drug release [70,71,72]. Natural polymers and polysaccharide-based hydrogels are excellent biomaterials due to their biocompatibility, but often exhibit poor mechanical properties. Conversely, synthetic polymer hydrogels can provide enhanced mechanical and viscoelastic properties, though they may show reduced biodegradability. Combining natural and synthetic polymers can yield hydrogels with moderate mechanical strength and good biodegradability, making them highly suitable as biomaterials [73]. Moreover, the incorporation of reactive oxygen species (ROS)-responsive polymeric nanoparticles into hyaluronic acid hydrogels has been shown to significantly enhance resistance to oxidative degradation. The excipient can serve as a beneficial ‘ROS sink’, protecting the HA matrix and prolonging hydrogel stability under oxidative conditions [74].

Although our experiments did not demonstrate a measurable extension of drug release with the incorporation of the additional polymer, the literature evidence suggests that modified HA-based hydrogels still offer significant advantages. Therefore, even in the absence of observed prolongation of release, incorporating an additional polymer may be advantageous for hydrogel stability, handling, and overall performance.

## 3. Materials and Methods

Disodium prednisolone 21-phosphate (PSP) was obtained from POL-AURA (Morąg, Poland). High-molecular-weight sodium hyaluronate (HA), with a molecular weight > 1.10 MDa, was purchased from ESCENT (Szczecin, Poland). Polyacrylic acid (Carbopol^®^ 980 NF, PA) was obtained from Lubrizol (Wickliffe, OH, USA), while ammonium acryloyldimethyltaurate/VP copolymer (Aristoflex^®^ AVC, AX) was supplied by Clariant International Ltd. (Muttenz, Switzerland). A polyvinyl acetate–polyvinylpyrrolidone mixture (PVA–PVP, Kollidon^®^ SR) was obtained from BASF (Ludwigshafen, Germany), and polyethylene glycol 4000 (PEG) was purchased from POL-AURA (Morąg, Poland). Trisodium phosphate dodecahydrate was sourced from CHEMPUR (Piekary Śląskie, Poland), and hydrochloric acid (35–38%) was supplied by Avantor Performance Materials (Piekary Śląskie, Poland). All chemicals were used as received without further purification. Semi-permeable cellulose membranes with a retention range of 5–8 μm were purchased from Carl Roth (Karlsruhe, Germany).

### 3.1. Hydrogels Preparation

An appropriate amount of PSP was dissolved in water. The resulting solution was then added to the required amount of HA or HA combined with an additional polymer. The mixture was homogenized using a homogenizer (Unidrive X 1000D, CAT, Staufen, Germany) until a uniform preparation was obtained. The composition of the formulations is presented in Table 1.

The hydrogels were stored for 24 h at 6 °C to remove air bubbles. The obtained preparations were then subjected to viscosity and release studies, after which they were left to dry at 6 °C. Subsequently, the dried formulations were analyzed using ATR–FTIR and DSC.

Physical mixtures were prepared by accurately weighing the individual components in the same ratios as used in the corresponding hydrogel formulations, followed by gentle manual mixing in a mortar to ensure homogeneity. No solvent or thermal treatment was applied during this process.

### 3.2. Viscosity Measurements

After 24 h storage at 6 °C, all hydrogel samples were visually inspected prior to viscometric measurements. No air bubbles were observed in the formulations at the time of testing. Therefore, no additional vacuum degassing step was performed before viscometric analysis. The spindle was carefully and slowly lowered into the hydrogel to minimize the risk of air bubble entrapment during sample loading. Each measurement was performed using a controlled and gentle loading procedure to ensure the integrity of the sample structure.

The viscosity study was carried out at 37 °C using a Brookfield rotation viscometer (DV2T, Brookfield, Middleboro, MA, USA) at a rotation speed of 50 rpm and with spindle No. 4 or No. 5. The viscosity of each formulation was measured six times, and the mean dynamic viscosity and the standard deviation was calculated. The dynamic viscosities of formulations F1–F5 were also compared using Student’s *t*-test.

### 3.3. ATR-FTIR Investigations

Attenuated Total Reflectance—Fourier Transform Infrared Spectroscopy (ATR–FTIR) was performed using an FTIR spectrometer operating in ATR mode (Nicolet iS50, Thermo Scientific, Waltham, MA, USA). Measurements were recorded at room temperature with a scan rate of 65 scans per minute. For each spectrum, 32 scans were collected in the wavenumber range of 4000–400 cm^−1^ at a resolution of 4 cm^−1^. ATR–FTIR spectra of the pure formulation components, the dried formulations F1–F5, and physical mixtures of their ingredients were recorded.

### 3.4. DSC Study

Differential Scanning Calorimetry (DSC) was performed using a differential scanning calorimeter (DSC 214 Polyma, Netzsch, Selb, Germany). Samples (3–5 mg) were placed in aluminum crucibles, covered with pierced lids, and sealed by pressing. Prior to DSC analysis, all hydrogel samples were pre-dried in a desiccator to minimize the influence of free water. Thermograms of the pure compounds, dried formulations F1–F5, and their physical mixtures were recorded over the temperature range of 0–300 °C at a heating rate of 5 °C min^−1^. This heating rate or even higher is commonly used as a compromise between sensitivity and experimental time for polymeric systems [75]. Nitrogen was used as the purge gas at a flow rate of 25 mL min^−1^.

### 3.5. Release Tests

Release measurements were carried out using paddle-over-disc apparatus (ERWEKA DT 126 Light, Heusenstamm, Germany) in accordance with Ph. Eur. 11.0 [76]. The hydrogel was placed in six donor chambers, covered with a cellulose membrane, and immersed in the acceptor fluid. The acceptor medium consisted of 1 L of phosphate buffer (pH = 6.8), reflecting the environment of the inflamed joint fluid [77]. The buffer was prepared using 0.1 mol/L HCl and 0.2 mol/L Na_3_PO_4_•12H_2_O, adjusted to pH 6.8 using 2 mol/L HCl or 2 mol/L NaOH resulting in an ionic strength of approximately 0.35–0.45 mol/L. The tests were performed at 37 °C with a rotational speed of 50 rpm. Samples of 3 mL were collected from the acceptor fluid at predetermined time intervals and immediately replaced with an equal volume of fresh medium. The drug concentration in the samples was determined spectrophotometrically using a UV–Vis spectrophotometer (JASCO V-530, Tokyo, Japan) at 248 nm, corresponding to its maximum absorbance. No interference from other hydrogel components was observed.

### 3.6. Dissolution Curves Comparison

Release curves, showing the relationship between the amount of drug released and time, were compared by calculating the difference factor (f_1_) and the similarity factor (f_2_) in accordance with FDA recommendations [55]. The equations have already been presented in our previous study [78]. The dissolution profiles were also compared using statistical methods including ANOVA and Student’s *t*-test.

### 3.7. Kinetic Calculations

The kinetics of PSP release from formulations F1-F5 were evaluated using zero-, first, and second-order kinetic models as well as Higuchi, Korsmeyer-Peppas and Peppas-Sahlin models. The equations for these models have been presented in our previous works [59,79]. Using the linear least-squares method, kinetic parameters such as release rate constants and half-release times were determined.

### 3.8. Statistical Analysis

Student’s *t*-test comparisons were performed using formulation F1 as the reference, as it did not contain any additional polymer. The dynamic viscosity values, kinetic parameters, coefficients of determination, and release exponents n and n’ are presented as mean values from six measurements, along with their standard deviations. All statistical analyses were conducted at a 95% confidence level.

## 4. Conclusions

The ATR–FTIR spectra, DSC thermograms, and viscometric measurements of formulations F1–F5 revealed interactions between PSP and the polymeric matrices. In F1, hydrogen bonding between PSP and HA was observed, likely present in other formulations as well. F2, containing PA, showed additional PSP–PA interactions, correlating with the highest dynamic viscosity and indicating the formation of a more extensive hydrogen-bonding network. In F3, PSP–AX interactions were suggested by spectral changes, explaining a moderate viscosity increase relative to F1, although the effect was weaker than in F2. No additional hydrogen bonding was detected between PSP and the PVA–PVP mixture in F4, consistent with similar viscometric behavior to F1. In F5, weak water-mediated interactions between PSP phosphate groups and PEG may account for the slight viscosity increase. The extent and strength of hydrogen-bonding interactions corresponded with viscosity trends. Despite these relations, PSP release profiles were similar across all hydrogels, indicating that the interactions are relatively weak and do not markedly influence drug transport. Increase in the concentration of additional polymers may strengthen the hydrogel network, potentially enabling more controlled PSP release.

## Figures and Tables

**Table 1:** The composition of the studied formulations.

Formulation	HA [%]	PSP [%]	PA [%]	AX [%]	PVA−PVP [%]	PEG [%]	Water [%]
F1	2.0	0.5	–	–	–	–	97.5
F2	2.0	0.5	0.5	–	–	–	97.0
F3	2.0	0.5	–	0.5	–	–	97.0
F4	2.0	0.5	–	–	0.5	–	97.0
F5	2.0	0.5	–	–	–	0.5	97.0

HA-sodium hyaluronate, PSP-prednisolone disodium phosphate, PA-polyacrylic acid, AX-ammonium acryloyldimethyltaurate/polyvinylpyrrolidone copolymer, PVA–PVP-polyvinyl acetate–polyvinylpyrrolidone mixture, PEG-polyethylene glycol.

**Table 2:** The difference factors f_1_ and the similarity factors f_2_ obtained from the comparison of the release curves, formulation F1 was set as a reference formulation.

f_1_				
formulation	F2	F3	F4	F5
F1	7.1	2.4	6.5	1.7
f_2_				
F1	74	91	75	94

**Table 3:** Kinetic parameters of PSP release from formulations F1–F5.

Kinetic Model	Kinetic Parameters	F1	F2	F3	F4	F5
Z-O	k_0_ × 10^2^ [mg × min^−1^]	7.6 ± 0.7	6.9 ± 0.6	7.0 ± 0.1	7.7 ± 0.1	7.5 ± 0.1
	t_0.5_ [min]	241.1 ± 22.5	274.0 ± 25.1	266.0 ± 25.3	245.5 ± 22.8	251.6 ± 22.1
	R^2^	0.94 ± 0.01	0.94 ± 0.01	0.94 ± 0.01	0.94 ± 0.01	0.95 ± 0.01
F-O	k_1_ × 10^3^ [min^−1^]	3.2 ± 0.1	2.5 ± 0.1	2.6 ± 0.1	3.0 ± 0.1	3.0 ± 0.1
	t_0.5_ [min]	216.5 ± 6.2	282.7 ± 11.1	270.7 ± 11.0	235.2 ± 7.7	233.1 ± 5.9
	R^2^	0.99 ± 0.01	0.99 ± 0.01	0.99 ± 0.01	0.99 ± 0.01	0.99 ± 0.01
S-O	k_2_ × 10^4^ [mg^−1^ × min^−1^]	2.1 ± 0.1	1.3 ± 0.3	1.4 ± 0.1	1.9 ± 0.1	1.7 ± 0.1
	t_0.5_ [min]	131.3 ± 7.6	209.3 ± 4.8	193.6 ± 4.8	142.0 ± 6.9	151.6 ± 7.6
	R^2^	0.98 ± 0.01	0.99 ± 0.01	0.99 ± 0.01	0.98 ± 0.01	0.98 ± 0.01
H	k_H_ [mg × min^−1/2^]	1.4 ± 0.3	1.2 ± 0.1	1.3 ± 0.1	1.4 ± 0.1	1.3 ± 0.1
	t_0.5_ [min]	183.4 ± 7.3	237.0 ± 8.6	222.3 ± 7.4	189.8 ± 6.8	200.8 ± 8.3
	R^2^	0.99 ± 0.01	0.99 ± 0.01	0.99 ± 0.01	0.99 ± 0.01	0.99 ± 0.01
K-P	k_K-P_ × 10^2^ [min^−n^]	2.9 ± 0.2	2.8 ± 0.2	2.9 ± 0.2	2.8 ± 0.2	2.5 ± 0.1
	t_0.5_ [min]	128.7 ± 19.8	131.4 ± 15.9	127.6 ± 15.4	128.7 ± 13.8	134.2 ± 13.4
	R^2^	0.99 ± 0.01	0.99 ± 0.01	0.99 ± 0.01	0.99 ± 0.01	0.99 ± 0.01
	n	0.59 ± 0.02	0.59 ± 0.01	0.58 ± 0.01	0.59 ± 0.01	0.60 ± 0.01
P-S	k_1P-S_ × 10^10^ [min^−n′^]	1.00 ± 0.01	1.00 ± 0.01	1.00 ± 0.01	1.00 ± 0.01	1.00 ± 0.01
	k_2P-S_ × 10^2^ [min^−n′^]	3.74 ± 0.01	3.52 ± 0.02	3.81 ± 0.01	3.66 ± 0.02	3.25 ± 0.02
	$n^{\prime }$	0.27 ± 0.02	0.27 ± 0.02	0.27 ± 0.01	0.27 ± 0.01	0.28 ± 0.01
best fit		F-O, H, K-P	F-O, S-O, H, K-P	F-O, S-O, H, K-P	F-O, H, K-P	F-O, H, K-P

Z-O-zero order, F-O-first order, S-O-second order, H-Higuchi, K-P-Korsmeyer-Peppas, P-S-Peppas-Sahlin.

## Data Availability

The data supporting the reported results are available in the Department of Physical Chemistry and Biophysics of Wroclaw Medical University.
